# Ketogenic Diet Therapy for Drug-Resistant Epilepsy and Cognitive Impairment in Children With Tuberous Sclerosis Complex

**DOI:** 10.3389/fneur.2022.863826

**Published:** 2022-05-24

**Authors:** Yu Fang, Dan Li, Man Wang, Xia Zhao, Jing Duan, Qiang Gu, Baomin Li, Jian Zha, Daoqi Mei, Guangbo Bian, Man Zhang, Huiting Zhang, Junjie Hu, Liu Yang, Lifei Yu, Hua Li, Jianxiang Liao

**Affiliations:** ^1^Shenzhen Children's Hospital, China Medical University, Shenzhen, China; ^2^Department of Pediatric, Second Affiliated Hospital of Xi'an, Jiaotong University, Xi'an, China; ^3^Epilepsy Center, Shanghai Neuromedical Center, Shanghai, China; ^4^Department of Neurology, Shenzhen Children's Hospital, Shenzhen, China; ^5^Department of Pediatric, First Hospital, Peking University, Beijing, China; ^6^Qilu Hospital, Shandong University, Jinan, China; ^7^Department of Neurology, Jiangxi Provincial Children's Hospital, Nanchang, China; ^8^Department of Neurology, Children's Hospital Affiliated to Zhengzhou University, Henan Children's Hospital, Zhengzhou Children's Hospital, Zhengzhou, China; ^9^Department of Pediatric Neurorehabilitation, People's Hospital of Ningxia Hui Autonomous Region, Yinchuan, China; ^10^Department of Neurology, Shantou University Medical College Shenzhen Children's Hospital, Shenzhen, China; ^11^Department of Pediatric, Guangdong Women and Children Hospital, Guangzhou, China; ^12^Department of Neurology, Children's Hospital of Fudan University, Shanghai, China; ^13^Department of Neurology, Guangdong 999 Brain Hospital, Guangzhou, China

**Keywords:** tuberous sclerosis complex, comorbidity, drug-resistant epilepsy, cognitive impairment, ketogenic diet, multi-center clinical trial, children

## Abstract

**Objective:**

Tuberous sclerosis complex (TSC) is a rare disease with a high risk of epilepsy and cognitive impairment in children. Ketogenic diet (KD) therapy has been consistently reported to be beneficial to TSC patients. In this study, we aimed to investigate the efficacy and safety of KD in the treatment of drug-resistant epilepsy and cognitive impairment in children with TSC.

**Methods:**

In this multicenter study, 53 children (33 males and 20 females) with drug-resistant epilepsy or cognitive impairment caused by TSC were retrospectively recruited from 10 hospitals from January 1, 2010, to December 31, 2020. Intention-to-treat analysis was used to evaluate seizure reduction and cognition improvement as outcomes after KD therapy.

**Results:**

Of the 53 TSC patients included, 51 failed to be seizure-free with an average of 5.0 (range, 4–6) different anti-seizure medications (ASMs), before KD therapy. Although the other two patients achieved seizure freedom before KD, they still showed psychomotor development delay and electroencephalogram (EEG) abnormalities. At 1, 3, 6, and 12 months after the KD therapy, 51 (100%), 46 (90.2%), 35 (68.6%), and 16 patients (31.4%) remained on the diet therapy, respectively. At these time points, there were 26 (51.0%), 24 (47.1%), 22 (43.1%) and 13 patients (25.5%) having ≥50% reductions in seizure, including 11 (21.6%), 12 (23.5%), 9 (17.6%) and 3 patients (5.9%) achieving seizure freedom. In addition, of 51 patients with psychomotor retardation, 36 (36 of 51, 70.6%) showed cognitive and behavioral improvements. During the KD therapy, no serious side effects occurred in any patient. The most common side effects were gastrointestinal disturbance (20 of 53, 37.7%) and hyperlipidemia (6 of 53, 11.3%). The side effects were gradually relieved after adjustment of the ketogenic ratio and symptomatic treatment.

**Conclusion:**

KD is an effective and safe treatment for TSC-related drug-resistant epilepsy and cognitive impairment in children. KD can reduce seizure frequency and may potentially improve cognition and behavior.

## Introduction

Tuberous sclerosis complex (TSC) is an autosomal-dominant multi-system neurocutaneous syndrome characterized by hamartomas involving the skin, central nervous system, heart, lungs, kidneys, and other organs. The estimated incidence of TSC is between 1:6000 and 1:10,000 in live births. TSC is mainly caused by mutations in *TSC1* at 9q34 or *TSC2* at 16p13 (1). The nervous system manifestations of TSC mainly include epilepsy, cognitive impairment, developmental delay, and other neurological defects, among which epilepsy is the most common (2, 3). In TSC patients, the incidence of epilepsy is 80–90%, with drug-resistant epilepsy accounting for 55–62% (4, 5). Mutations in the *TSC1* or *TSC2* gene can lead to the over-activation of the mammalian target of rapamycin (mTOR) signaling pathway, resulting in subcortical tubers, which are the leading cause of seizures in TSC (6). Moreover, multidrug-resistant proteins and related genes, such as multidrug resistance type 1 gene and multidrug resistance-associated protein-1 transporters, are widely expressed in subcortical tubers (7, 8).

Tuberous-sclerosis-associated neuropsychiatric disorders (TAND) is an umbrella term encompassing the full range of neurodevelopmental, behavioral, psychiatric, and psychosocial manifestations associated with TSC (9). Almost all patients with TSC would have some of these neuropsychiatric manifestations in their lifetime (10). The common neurodevelopmental disorders associated with TSC are autism spectrum disorders (ASDs) (40–50%) and attention deficit hyperactivity disorder (30–50%) (11–13). Tubers have traditionally been considered as the critical pathological substrate: the tubers can directly cause seizures and the tuber load correlates with intellectual disability and autism (14). Recently, studies in knockout mice with *TSC* gene inactivation specifically in glial cells have shown cell-autonomous effects within glia. Different types of glial cells have emerged as major contributors to TAND and other neurological phenotypes of the genetic disorder TSC (15).

The ketogenic diet (KD) is a high-fat, low-carbohydrate, adequate-protein diet, with additional adequate, balanced nutrients (16). KD was first used as a therapeutic method for epileptic seizures in 1921 and was introduced into China in 2004 (17). Current studies suggest that the KD can play an anti-epileptic role by inhibiting the over-activated mTOR signaling pathway and through other multi-target mechanisms involving neurotransmitters, brain energy metabolism, oxidative stress, and ion channels (18–20). Ketone bodies (KBs) have recently been reported to act as neuroprotective agents by increasing ATP levels and reducing the production of reactive oxygen species in neurological tissues, together with increased mitochondrial biogenesis, which may enhance the regulation of synaptic function (21). Moreover, increased brain ketone uptake is positively related to episodic memory, language, executive function, and processing speed (22). Although successful treatment of TSC-related epilepsy and cognitive improvement by KD has been reported in recent years, there is still a lack of sufficient data on seizure reduction and cognitive improvement, particularly multi-center data in children. In this study, we conducted a multi-center retrospective study to analyze the efficacy and safety of KD in the treatment of TSC-related drug-resistant epilepsy and cognitive impairment in children with TSC, to improve the prognosis in children with TSC.

## Materials and Methods

### Patients

Fifty-three children with cognitive impairment or drug-resistant epilepsy caused by TSC were retrospectively enrolled. They received KD therapy at Shenzhen Children's Hospital, Shanghai Neuromedical Center, Second Affiliated Hospital of Xi'an Jiaotong University, Guangdong 999 Brain Hospital, Children's Hospital of Fudan University, Shandong University Cheeloo College of Medicine, Peking University First Hospital, People's Hospital of Ningxia Hui Autonomous Region, Jiangxi Provincial Children's Hospital, or Children's Hospital Affiliated to Zhengzhou University, from January 1, 2010, to December 31, 2020. The patients were recruited according to the following inclusion criteria. First, the patients should meet the diagnostic criteria of TSC following the International TSC Consortium in 2012 and 2018 (23, 24); that is, patients having a pathogenic variant in *TSC1* or *TSC2*. For patients not having genetic test reports, clinical criteria (the presence of two major criteria or one major and two minor criteria) of TSC should be met. Second, the patients should be diagnosed with drug-resistant epilepsy, i.e., failure to achieve sustained seizure freedom after adequate trials of two tolerated, appropriately chosen anti-seizure medications (ASMs) schedules (whether as monotherapies or in combination) (25). Patients diagnosed with drug-resistant epilepsy may have failed to respond to epilepsy surgery or did not receive surgery evaluation before KD. Besides, TSC patients with West syndrome or infantile spasms who failed to respond to adrenocorticotropic hormone and another ASM were also recruited. Third, two patients still having an abnormal electroencephalogram (EEG) discharge or global development delay after administration of two or more kinds of ASM schedules were also included. Abnormal EEG refers to EEG with the presence of epileptic discharges. Global development delay was defined as motor and mental development lagging behind normal children of the same age. The intelligence quotient (IQ) and developmental quotient (DQ) tests were not commonly conducted, only 14 patients had the tests before KD, and four had the tests after KD. Development delay was diagnosed mainly based on a thorough clinical history and a detailed physical examination by a trained specialist. The 51 patients were diagnosed with psychomotor development delay at KD initiation, and three of them were diagnosed with ASD. At last, the patients were 0.4–14 years of age and had a KD treatment and follow-up time of ≥1 month. To avoid the influence of possible late response, we used the efficacy at 3 months after KD to the analysis of the KD's effect on reducing seizures and improving cognition and behavior. The exclusion criteria for patients were: (1) patients having severe diseases of vital organs, such as severe hepatic, renal or cardiac insufficiency, and immune deficiency; and (2) children lacking major medical records after KD and whose parents disagreed to participate in this study.

### Study Design

#### Implementation of KD

The patients initiated KD as inpatients. 50 patients initiated the classic KD, of whom 30 patients initiated KD with a ketogenic ratio (the ratio of fat to carbohydrate and protein) of 2:1, 15 with 4:1, 4 with 3:1, and 1 with 2.5:1. While the other 3 patients were treated with a modified Atkins diet (MAD), of which the lipid-to-nonlipid ratio was not strict, ranging from 1:1 to 1.5:1, but carbohydrates were kept at 10–20 g/day. During the maintenance period of KD therapy, 43 patients continued the classical KD, in which 29 with 1.5–4 :1, 9 with 4:1, 4 with 2:1, and 1 with 3:1 ketogenic ratio, while 10 patients received the MAD. Before KD initiation, the dietitians designed the KD meal plans based on the patients' food habits and body weights. During the KD treatment, other diets were stopped, and the original ASMs did not change within 3 months after KD initiation. Meanwhile, potassium citrate, multi-vitamins, essential minerals, and calcic agent without sucrose and lactose were supplemented in the daily diet. For the first week of KD, all patients were inpatients and were closely monitored for any possible adverse effect, and their parents or caregivers were trained on how to calculate the dietary ratio and make ketogenic foods at home. After discharge, the patients were asked to make daily records of seizures, calories, KD foods, and side effects. At 1, 2, 3, and 6 months after KD, the patients revisited the hospitals. After 6 months of KD, the revisit interval was extended to half a year if the KD was effective. In addition, the dietitians followed up with the patients by telephone or WeChat monthly. In any emergency, patients could go to the nearest hospital or contact doctors and dietitians in the KD group.

#### Data Collection

A unified information collection form was developed to collect data on clinical manifestations, cognitive and behavioral status, ASMs, and seizure frequency before and after KD. Information on the age of KD initiation, dietary ketogenic ratio, duration, efficacy, and adverse effects of KD therapy was also collected *via* this form. The seizure types were classified following the 2017 International League against Epilepsy classification criteria (26). At KD initiation, the parents or caregivers received specialized training from neurologists on identifying the seizure type and frequency and assessing psychomotor developmental issues during the hospital stay. Correlations between TSC genotype and KD efficacy were assessed based on the results of whole-exome sequencing. Information of cortical tubers, subependymal nodules, and subependymal giant cell astrocytoma confirmed by magnetic resonance imaging (MRI) was also collected.

#### KD Efficacy Analysis

KD efficacy was evaluated in terms of seizure reduction and psychomotor development. For assessment of seizure reduction, baseline (Pre-KD) seizure frequency was recorded by parents or guardians 1 month before initiation of KD, defined as the total seizure frequency during the 1 month before the initiation of KD. Patients with ≥50% reduction in total seizures during a particular month after KD were defined as responders to KD. Seizure freedom after KD was defined as the absence of seizures for at least one month. As the developmental test is relatively scarce, only 14 patients underwent the test before KD, while 4 had the test after KD. The psychomotor improvement was assessed based on pediatric neurologists' examination and caregiver' evaluation at 3 months after KD. For development evaluation by family members, the evaluation was made based on language expression, instruction execution, and learning ability. There were three levels of cognitive and behavioral improvement: Grade I for obvious improvement, Grade II for no obvious change, and Grade III for regression in cognition and behavior. Patients with obvious improvement (Grade I) were defined as responders to KD.

#### KD Safety Analysis

Blood and urine routine examination, liver and kidney function, blood electrolytes, blood lipids, gallbladder, and urinary system color ultrasound examinations were conducted at baseline, and at 1, 3, 6, and 12 months after KD. In addition, any adverse clinical events presumed to be related to KD were recorded.

### Data Analysis

We used intention-to-treat analysis to assess the outcomes in terms of seizure reduction, development, and cognition improvement. The Chi-square test was used to analyze the effects of sex and cortical tubers on the efficacy of KD. Fisher's exact probability method was used to analyze the effects of seizure type, genotype, and dietary ketogenic ratio on the efficacy of KD. The nonparametric test was used to analyze the effects of the age at epilepsy onset, the age at KD initiation, or the duration of epilepsy before KD. Paired *t*-test was used to analyze the change of ASMs pre and after KD. Comparisons were considered statistically significant when p-values were below 0.05 in a univariate test or below corrected p-value (corrected p-value = 0.05/n, n is the times of comparisons) by Bonferroni in multiple univariate tests. Data analyses were performed using the IBM SPSS statistics 25.0 software.

### Ethics Approval, Clinical Registrations, and Patient Consents

This study was approved by the Ethics Committee of Clinical Research of Shenzhen Children's Hospital (permission number, 2021010). The study was registered with the Chinese Clinical Trial Registry, and the registration number is ChiCTR2100047909. All guardians of patients consent for this retrospective analysis and publication of information relating to them.

## Results

### Patient Characteristics

Demographics and epileptic characteristics of the included patients are presented in Table 1. There were 33 males and 20 females. Thirty-four patients had genetic testing. *TSC1* gene mutation was found in 6 patients and *TSC2* in 24 patients, 4 with negative results. Fifty-one patients were diagnosed with psychomotor retardation. The mean age of epilepsy onset was 6.0 (4–16) months, and the mean duration of epilepsy before KD was 22.7 (12–41) months. Each patient suffered 2.0 (1–3) types of seizures on average. In addition, 22 patients (41.5%) were diagnosed with West syndrome, one (1.9%) with Lennox-Gastaut syndrome. There were 30 patients (56.6%) diagnosed with non-syndromic epilepsies, in whom 17 patients with focal onset, six patients with tonic seizures, five patients with epileptic spasms, one patient with the tonic-clonic seizure, and another patient with myoclonic seizure. An average of 5.0 (4–6) ASMs were used among the 53 patients before KD, two 2 of them experienced seizure freedom before KD initiation but had psychomotor development retardation and EEG abnormalities. A total of 29 patients (54.7%) were treated with mTOR inhibitors, three patients (5.6%) with Everolimus, and 26 patients (49.1%) with Sirolimus. The mean age at KD initiation was 40.0 (20–57) months, and the mean duration of KD was 34.7 (17.4–56.46) weeks, by December 31, 2020.

**Table 1:** Patient characteristics.

**Characteristics**	**Total (*n* = 53)**
**Gender**, ***n*** **(%)**	
Male	33 (62.3%)
Female	20 (37.7%)
**TSC gene**, ***n*** **(%)**	
TSC1 gene mutation	6 (11.3%)
TSC2 gene mutation	24 (45.3%)
TSC genetic test with negative results	4 (7.5%)
Not tested	19 (35.8%)
**Characteristics of head MRI**, ***n*** **(%)**	
Cortical tubers	35 (66.0%)
Subependymal nodules	39 (73.6%)
Subependymal giant cell astrocytoma	1 (1.9%)
**Age at onset of epilepsy, median (IQR** ^**a**^ **), months**	6.0 (4–16)
**Psychomotor development at KD initiation**, ***n*** **(%)**	
Normal	2 (3.8%)
Delayed	51 (96.2%)
**Seizure type at KD initiation**, ***n*** **(%)**	
Focal onset	20 (37.7%)
Generalized onset	
Epileptic spasms	19 (35.8%)
Tonic	7 (13.2%)
Tonic-clonic	2 (3.8%)
Clonic	1 (1.9%)
Myoclonic	1 (1.9)
Atonic	1 (1.9%)
Seizure-free	2 (3.8%)
**Epilepsy syndrome prior to KD**, ***n*** **(%)**	
West syndrome	22 (41.5%)
Lennox-Gastaut syndrome	1 (1.9%)
Non-syndromic epilepsy	30 (56.6%)
**Number of ASMs used prior to KD, median (IQR)**	5.0 (4–6)
**Epilepsy duration prior to KD, median (IQR), months**	22.7 (12–41)
**Age at KD initiation, median (IQR), months**	40.0 (20–57)
**Duration of KD, median (IQR), weeks**	34.7 (17.4–56.46)

a *IQR: interquartile range (25–75 percentiles)*.

### Response to KD

#### The Response Rate to KD in Terms of Seizure Reduction, Overall and at Each Time Point

For the 51 patients who did not achieve seizure freedom after use of ASMs, 51 (100%), 50 (98.0%), 46 (90.2%), 35 (68.6%), and 16 (31.4%) patients of them remained on the diet at 1, 2, 3, 6 and 12 months after KD, respectively, and 26 (51.0%), 23 (45.1%), 24 (47.1%), 22 (43.1%), and 13 (25.5%) of them experienced ≥50% reduction of seizures at the corresponding time points. In addition, 11 (21.6%), 13 (25.5%), 12 (23.5%), 9 (17.6%), and 3 (5.9%) patients achieved seizure freedom at 1, 2, 3, 6, and 12 months after KD, respectively (Table 2).

**Table 2:** Response rate in seizure reduction at 1, 2, 3, 6, and 12 months after KD (*n*, %).

**Reduction in seizures**	**1 month after KD**	**2 months after KD**	**3 months after KD**	**6 months after KD**	**12 months after KD**
Seizure-free	**11 (21.6)**	**13 (25.5)**	**12 (23.5)**	**9 (17.6)**	**3 (5.9)**
Reduced by 90~99%	1 (2.0)	0	2 (3.9)	3 (5.9)	4 (7.8)
Reduced by 50~90%	14 (27.4)	10 (19.6)	10 (19.6)	10 (19.6)	6 (11.8)
Reduced by <50%	13 (25.5)	15 (29.4)	12 (23.5)	6 (11.8)	0
No reduction	12 (23.5)	12 (23.5)	10 (19.6)	7 (13.7)	3 (5.9)
Total	51 (100)	50 (98.0)	46 (90.2)	35 (68.6)	16 (31.4)

#### Efficacy in Improving Cognition and Behavior

Before the initiation of KD, 51 of the 53 patients were diagnosed with psychomotor retardation, and two of the 51 patients had achieved seizure freedom before KD. At 3 months after KD, 36 patients (36/51, 70.6%) had obvious improvement in cognition and behavior assessed by neurologists' physical examination and parents' comprehensive judgment based on language expression, instruction execution, and learning ability (Table 3). In particular, at 3 months after KD, 3 patients (3/51, 5.9%), who were diagnosed with ASD before KD, were reported with an obvious improvement in behavior assessed by caregivers and doctors based on concentration and learning abilities, and social behavior and interactions of children.

**Table 3:** Psychomotor improvement at 3, 6, and 12 months after KD (*n*, %).

**Items**	**3 months after KD**	**6 months after KD**	**12 months after KD**
Number of patients remaining on KD	48 (94.1)	36 (70.6)	20 (39.2)
Psychomotor improved	**36 (70.6)**	29 (56.9)	17 (33.3)

Of the 49 patients diagnosed with psychomotor retardation and had seizure onset at the initiation of KD, 34 (34/49, 69.4%) had cognition and behavioral improvement at 3 months after KD. In addition, 26 patients (26/34, 76.5%) experienced ≥50% reduction in seizures, including 10 patients (10/34, 29.4%) with seizure freedom. As for ASMs, the 29 patients with psychomotor improvement at 6 months after KD, using 3.17±1.07 different ASMs, compared to 3.38±1.21 at the initiation of KD (*P*=0.161). The 17 patients with psychomotor improvement at 12 months after KD, using 3.06 ± 1.34 different ASMs, compared to 3.24 ± 1.39 at the initiation of KD (*P* = 0.455). Ten patients (10/36, 27.8%) continued to use mTOR inhibitors from KD initiation to 6 months after KD.

#### Comparison of Seizure Reduction Between the Responders and the Non-responders

For the 51 patients with seizure onset at the initiation of KD, at 3 months of KD, 46 patients remained on KD, including 24 responders and 22 non-responders. There was no statistical significant difference in age of epilepsy onset (*P* = 0.349), age of KD initiation (*P* = 0.531), epilepsy duration before KD (*P*=0.138), cortical tubers (*P* = 0.686), or sex (*P* = 0.958) between the two groups.

We also assessed the correlation between TSC genotype and KD efficacy. At 3 months after KD, there were two patients (2/6, 33.3%) with *TSC1* gene mutation and nine patients (9/22, 40.9%) with *TSC2* gene mutation experienced ≥50% reduction in seizures, respectively. There was no statistical significant difference in KD efficacy among groups with *TSC1* and *TSC2* gene mutations (*P* = 0.622).

#### KD Efficacy in Groups of Different Seizure Types at KD Initiation

In the first month of KD, none of the patients withdrew from KD. The proportions of KD responders in children with seizure types of focal onset, epileptic spasms, tonic, and clonic seizures were 60.0, 52.6, 42.9, and 100.0%, respectively. At the same time, four patients (20.0%) with the focal onset and seven patients (36.8%) with epileptic spasms experienced seizure freedom. However, two patients with generalized tonic-clonic seizures, one patient with myoclonic seizures, and one patient with tonic seizures did not significantly benefit. None of the patients with tonic or clonic seizures could achieve seizure free. There was no significant difference in KD efficacy among patients with different seizure types at KD initiation (*P* = 0.490).

#### KD Efficacy in Patients With Different Ketogenic Ratios

At the first month of KD, the proportions of responders with ketogenic ratios of 2:1, 4:1, 3:1, 1:1, and 2.5:1 were 60.0, 46.2, 0.0, 33.3, and 100.0%, respectively. There were 6 (20.0%) and 5 patients (38.5%) with seizure freedom for at least 1 month in the 2:1 and 4:1 groups, respectively, while none achieved seizure freedom in the 3:1, 1:1, and 2.5:1 groups (Table 4). There was no significant difference in the efficacy of KD among groups of different ketogenic ratios at initiation (*P* = 0.133).

**Table 4:** KD efficacy in groups of different ketogenic ratios at initiation.

**Ketogenic ratio at KD initiation**	**Enrolled**	**Responded**	**No effect**	**Seizure freedom**
	***n* (%)**	***n* (%)**	***n* (%)**	***n* (%)**
2:1	30 (100.0)	**18 (60.0)**	12 (40.0)	**6 (20.0)**
4:1	13 (100.0)	**6 (46.2)**	7 (53.8)	**5 (38.5)**
3:1	4 (100.0)	0	4 (100.0)	0
1:1	3 (100.0)	**1 (33.3)**	2 (66.7)	0
2.5:1	1 (100.0)	**1 (100.0)**	0	0

At the third month of KD, the proportions of responders in children with maintenance ketogenic ratios of 1.5–4:1, 4:1, 2:1, 3:1 and 1–1.5:1 were 46.4%, 25.0%, 75.0%, 0.0%, and 60.0%, respectively. There were 5 (17.9%), 2 (25.0%), 1 (25.0%), and 4 patients (40.0%) with seizure freedom in the groups with ketogenic ratios of 1.5–4:1, 4:1, 2:1, and 1–1.5:1, respectively (Table 5). There was no significant difference in KD efficacy among the groups of different ratios during the maintenance period (*P* = 0.493).

**Table 5:** KD efficacy in groups of different ketogenic ratios in the maintenance period.

**Ketogenic ratio in the maintenance period**	**Enrolled**	**Remained**	**Responded**	**No effect**	**Seizure freedom**
	***n* (%)**	***n* (%)**	***n* (%)**	***n* (%)**	***n* (%)**
1.5~4:1	28 (100.0)	26 (92.9)	**13 (46.4)**	13 (46.4)	**5 (17.9)**
4:1	8 (100.0)	6 (75.0)	**2 (25.0)**	4 (50.0)	**2 (25.0)**
2:1	4 (100.0)	4 (100.0)	**3 (75.0)**	1 (25.0)	**1 (25.0)**
3:1	1 (100.0)	1 (100.0)	0	1 (100.0)	0
1–1.5:1(MAD)	10 (100.0)	9 (90.0)	**6 (60.0)**	3 (30.0)	**4 (40.0)**

### Safety of KD Therapy

During the KD treatment, none of the 53 patients reported severe side effects. The main side effect was gastrointestinal disturbance (20/53, 37.7%), including abdominal pain, vomiting, diarrhea, and constipation. The second most common side effect was hyperlipidemia (6/53, 11.3%), leading to total cholesterol level ≥5.18 mmol/L (200 mg/dl) and/or triglyceride level ≥1.70 mmol/L (150 mg/dl) in plasma. The third most common side effect was infection (3/53, 5.7%), including one patient with pneumonia caused by severe coughing and two patients with upper respiratory tract infection. Other side effects included kidney stones (2/53, 3.8%), slow growth (2/53, 3.8%), hypoproteinemia (1/53, 1.9%), and hyperuricemia (1/53, 1.9%) (Table 6). All of the above side effects were relieved after adjustment of the ketogenic ratio and symptomatic treatment.

**Table 6:** Adverse effects reported during the KD treatment.

**Adverse effect**	***n* (%)**
No adverse effect	30 (56.6)
**Gastrointestinal disturbance**	**20 (37.7)**
**Hyperlipidemia**	**6 (11.3)**
Infectious disease	3 (5.7)
Kidney stones	2 (3.8)
Slow growth	2 (3.8)
Hypoproteinemia	1 (1.9)
Hyperuricemia	1 (1.9)

## Discussion

In our study, we evaluated the efficacy and safety of KD in the treatment of drug-resistant epilepsy and cognitive impairment related to TSC in children. The overall response rates in terms of seizure reduction at 1, 2, 3, 6, and 12 months after KD were 51.0, 45.1, 47.1, 43.1, and 25.5%, respectively. In addition, 36 of the 51 patients (70.6%) with psychomotor retardation exhibited obvious improvement of cognitive function after KD therapy.

The efficacy of KD for TSC-associated epilepsy has previously been evaluated in single-center or smaller sample-size studies. For example, Kossoff et al., (5) first reported that at 6 months after starting KD, 11 of 12 children with TSC (92%) experienced >50% reduction of seizures. Park et al. (27) analyzed 12 children with drug-resistant epilepsy related to TSC who received KD therapy and found that at 3 months of KD treatment, 10 patients (83.3%) had a >50% reduction of seizures. Youn et al. (28) also studied the long-term efficacy of KD for drug-resistant epilepsy in patients with TSC and found that at 3 months of KD, 21 of 31 patients (67.7%) had >50% reduction of seizures. All these studies suggest that KD may be effective in the treatment of drug-resistant epilepsy associated with TSC; however, more studies on the application of KD in this rare disease group are needed.

The findings of cognitive improvement in this multicenter study are similar to the results of a single-center study by Park et al. (27), which showed that in 12 patients with TSC-related epilepsy, four patients (33.3%) were “much improved” and five patients (41.7%) were “somewhat improved” after 3 months of KD. In particular, there are three patients (3/51, 5.9%) diagnosed with ASD before KD, were reported with an obvious improvement in behavior, after receiving KD therapy for a mean duration of 20.2 (17.37 to 26) weeks. Therefore, KD might be an effective therapy for cognitive impairment and ASD in TSC.

During KD therapy, KBs replace glucose to be the main brain fuel. This process benefits the potassium channel, which is sensitive to adenosine triphosphate, adenosine, and γ-aminobutyric acid (GABA) energy activity; increases the expression of brain-derived neurotrophic factor; expands energy reserves; and improves mitochondrial function, ensuring stabilization of neuron action potential (21, 29). Ketone substrates also improve the structural and functional synaptic plasticity and lead to the activation of the signaling pathway that reinforces neural bioenergy and resistance to oxidative stress (30, 31). The benefits of cognition can be translated into improvements in verbal and recognition memory, verbal fluency, executive function, and global cognition (21, 32, 33). Nevertheless, further research is needed to clarify the mechanisms by which KD improves cognition and behavior function of patients with developmental delay and ASD.

Although patients with psychomotor improvement did not reduce ASMs at 6 and 12 months after KD (*P* = 0.161 and *P* = 0.455, respectively), some patients with seizure freedom did have a reduction in the burden of ASMs. In our study, 18 patients (18/51, 35.3%) experienced seizure freedom, and four of the 18 patients (4/51, 7.8%) tried to reduce the use of ASMs; among them, three patients (3/51, 5.9%) still maintained seizure freedom, but one patient (1/51, 2.0%) experienced seizure recurrence. In addition, one patient treated with KD who experienced >90% reduction of seizures maintained the state of >90% reduction of seizures after reducing ASMs. Therefore, KD is beneficial in some children with drug-resistant epilepsy related to TSC, when the use of ASMs is reduced, which is similar to the conclusion of Youn et al. (28). When KD treatment fails, surgery may be an alternative option, and one patient in our study chose surgery after KD failure.

Consistent with the report by Youn et al. (28), our study showed that there was no direct correlation between KD efficacy and seizure type at KD initiation, *TSC* gene mutation, or the interval from seizure onset to KD initiation. Meanwhile, the efficacy of KD in the treatment of drug-resistant epilepsy associated with TSC was not affected by the age of epilepsy onset, age at KD initiation, sex, cortical tubers, or the ketogenic ratio. However, Youn et al. (28) found that the ages at seizure onset and KD initiation were significantly earlier in patients who experienced a recurrence of seizures after reaching seizure freedom than in patients with sustained seizure freedom (*P* = 0.005 and *P* = 0.005, respectively). Furthermore, patients who experienced a recurrence of seizures after seizure freedom were treated with significantly more ASMs than patients with sustained seizure freedom (*P* = 0.009).

Another issue is the relationship between the ketogenic ratio and KD efficacy. In our study, there was no significant difference in KD efficacy either among groups of different ketogenic ratios at KD initiation (*P* = 0.133) or among groups of different ketogenic ratios during the maintenance period (*P*= 0.493). However, a previous report by Seo et al. (34) showed that the 4:1 KD had greater antiepileptic efficacy than the 3:1 KD (*P* < 0.05) at 3 months after initiating the diet. Bough et al. (35) also found that the seizure threshold was significantly elevated with increased ketogenic ratios in rats. The discrepancies between our study and the above studies may be due to the following reasons. First, the sample size in each ketogenic ratio group was too small. Second, the lack of association of ketogenic ratio at KD initiation with the efficacy in the first month after KD may be because the time was too short for patients to show response. Third, the lack of effect of ketogenic ratio in the maintenance period on the KD efficacy in the third month after KD maybe because most (26/37, 70.3%) patients using the classical KD had adjusted the ketogenic ratio (1.5–4:1) during the maintenance period, while a few (11/37, 29.7%) patients used a fixed ratio (4:1 or 3:1 or 2:1) during the maintenance period.

The mTOR signaling pathway plays a crucial role in brain development, and the TSC-related neuropsychological abnormalities are related to the over-activation of the mTOR signaling pathway (10, 36). McDaniel et al. have confirmed that KD could inhibit the over-activation of the mTOR signaling pathway in animal models (37). In addition, Warren et al. (20) confirmed that decanoic acid, a vital component of the medium-chain triglyceride KD, could decrease the mTORC1 activity in rat hippocampus *ex vivo* and TSC patient-derived astrocytes. These results provide a biological mechanism of KD efficacy in TSC. However, in the case series of five TSC patients, KD did not induce tumor regression or suppress the growth of TSC-related tumors (38). KD did not appear to be able to provide the same level of mTOR inhibition required to cause tumor regression. The exact mechanism of KD-mediated improvement of seizures and cognitive behavior in TSC patients is still not fully understood and needs further study.

There are several limitations to our study. First, this was a retrospective study, so the data was not complete enough. In our study, for the lack of IQ and DQ tests, the evaluation of psychomotor improvement was mainly based on subjective assessment by neurologists and parents. Future studies are needed to compare psychomotor states before and after KD by some objective assessment such as formal IQ and DQ tests, and formal questionnaires of assessment of life quality or even childhood autism rating scale in TSC with ASD. Second, since the follow-up time of this study was 12 months, we mainly analyzed the short-term efficacy of KD in the treatment of TSC. More studies are needed to explore the long-term outcomes of KD in the future. Third, we did not analyze the relationship between KBs and KD efficacy, although KBs were regularly monitored in children on KD. Fourth, due to the limited medical resources, we did not perform enough EEG recording during the KD. The cycle of EEG reexamination in children with epilepsy is usually 6 to 12 months, so it is difficult to perform follow-up EEG every 1 to 2 months after KD initiation in clinical practice. Therefore, we should continue collecting the relevant data to further explore the long-term outcomes in the future.

## Conclusions

KD is an effective and safe treatment for children with TSC-related drug-resistant epilepsy and cognitive impairment. KD can reduce seizure frequency and may potentially improve cognition and behavior.

## Data Availability Statement

The original contributions presented in the study are included in the article/Supplementary Material, further inquiries can be directed to the corresponding authors.

## Ethics Statement

The studies involving human participants were reviewed and approved by the Ethics Committee of Clinical Research of Shenzhen Children's Hospital. Written informed consent to participate in this study was provided by the participants' legal guardian/next of kin.

## Author Contributions

YF, JL, LY, and HL designed the study. YF drafted the article. All authors analyzed and interpreted the clinical and diagnostic data, critically reviewed the manuscript, and read and approved the final manuscript.

## Funding

This work was supported by the Sanming Project of Medicine in Shenzhen (No. SZSM201812005), the Shenzhen Key Medical Discipline Construction Fund (No. SZXK033), the Shenzhen Fund for Guangdong Provincial Highlevel Clinical Key Specialties (No. SZGSP012), the Shenzhen Fund (No. JCYJ20200109150818777), and the Joint Construction Project of Medical Science and Technology in Henan Province (No. LHGJ20200618).

## Conflict of Interest

The authors declare that the research was conducted in the absence of any commercial or financial relationships that could be construed as a potential conflict of interest.

## Publisher's Note

All claims expressed in this article are solely those of the authors and do not necessarily represent those of their affiliated organizations, or those of the publisher, the editors and the reviewers. Any product that may be evaluated in this article, or claim that may be made by its manufacturer, is not guaranteed or endorsed by the publisher.

## Abbreviations

TSC: Tuberous sclerosis complex
KD: ketogenic diet
KBs: Ketone bodies
mTOR: mammalian target of rapamycin
ASM: anti-seizure medication
EEG: electroencephalogram
ASD: autism spectrum disorder
IQR: interquartile range (25–75 percentiles)
IQ: intelligence quotient
DQ: developmental quotient.
